# Establishment and identification of an animal model of long-term exercise-induced fatigue

**DOI:** 10.3389/fendo.2022.915937

**Published:** 2022-08-26

**Authors:** Kai Yan, Haoyang Gao, Xiaohua Liu, Zhonghan Zhao, Bo Gao, Lingli Zhang

**Affiliations:** ^1^ School of Kinesiology, Shanghai University of Sport, Shanghai, China; ^2^ Institute of Orthopaedic Surgery, Xijing Hospital, Fourth Military Medical University, Xi’an, China; ^3^ College of Athletic Performance, Shanghai University of Sport, Shanghai, China

**Keywords:** exercise-induced fatigue, treadmill, long-term, swimming, multiple organ phenotype, bone

## Abstract

In competitive sports, the training load is close to the human physiological limit, which will inevitably lead to exercise-induced fatigue. If fatigue cannot be recovered in time, it will eventually lead to excessive training and affect sport performance. Therefore, fatigue has become an important part of the physical function assessment for athletes. This paper will review animal models of long-term exercise-induced fatigue, modeling schemes of mice under treadmill and swimming training, phenotypes of long-term exercise-induced fatigue (e.g., nervous system damage, myocardial cell damage, bone mineral density changes, and skeletal muscle damage), and fatigue indicators. The relationship between physiological indicators and biomarkers and long-term exercise-induced fatigue is analyzed to promote exercise-induced fatigue monitoring. This paper attempts to provide a reference for the selection of animal models of long-term exercise-induced fatigue and provide a new theoretical basis for medical supervision and recovery of exercise-induced fatigue.

## Introduction

Exercise-induced fatigue is a complex physiological phenomenon. After exercising for a certain period of time while sustaining a given intensity, athletic ability and organ function temporarily decline and can only be restored after appropriate time to rest and adjustment (1). Exercise-induced fatigue is also when the body cannot sustain its function at a specific level or maintain a predetermined exercise intensity, that is, an inability to complete a task that was once achievable within a recent time frame (2). If exercise-induced fatigue continue to increase, it will lead to endocrine system dysfunction, decreased immunity, insomnia, depression, and other adverse physiological changes (3, 4). Fatigue in sports also affects performance and causes serious injuries (5).

There are two different classifications of exercise-induced fatigue in the human body: physiological and psychological. These are distinguished by triggering mechanism and fatigue performance. Physiological fatigue is manifested by a decline in motor ability, while psychological fatigue is manifested by a behavioral change. According to their etiology, exercise-induced fatigue is mainly divided into central fatigue and peripheral fatigue. They come from two main paths: one is through the central nervous system, and the other is through the peripheral nervous system involving muscles (6). Exercise-induced fatigue can be divided into transient exercise-induced fatigue and long-term exercise-induced fatigue according to its duration. Transient exercise-induced fatigue occurs after short-term, high-intensity exercise. The main physiological features of fatigue are inhibition of neuroendocrine and hematopoietic system functions and decrease in immune function and anti-peroxidation ability of the body (7). Transient fatigue needs to be monitored in a timely manner to avoid the accumulation of long-term exercise-induced fatigue, which has adverse effects on athletic ability and athletes.

Fatigue is a gradual process. Within a certain range, the body can eliminate fatigue after exercise, restore function, and prepare for the next exercise. If excessive exercise continues and recovery time is insufficient, fatigue will accumulate due to inadequate recovery (7). Long-term exercise-induced fatigue occurs after long-term, continuous, high-intensity exercise exceeds the body’s natural recovery function. The main reasons for long-term exercise-induced fatigue are glucose storage, muscle glycogen exhaustion, and nerve–endocrine–immune-system disorder. Exercise immunosuppression refers to the phenomenon that excessive exercise leads to the decline in immune function, which also affects the body’s ability to recover (8). Exhaustion is a special form of fatigue and the final stage of fatigue development. Therefore, subjects in fatigue experimental studies often target exhaustion as the end of their exercise rather than voluntary termination (9).

In this paper, we summarize long-term exercise training for fatigue models in experimental animals. Therefore, the establishment of animal models of long-term exercise-induced fatigue needs to be standardized in literature. At the same time, long-term exercise-induced fatigue can occur in different organs. Fatigue indicators have become an important factor in guiding animal modeling.

## Establishment of long-term exercise-induced fatigue animal model

**Figure 1:**
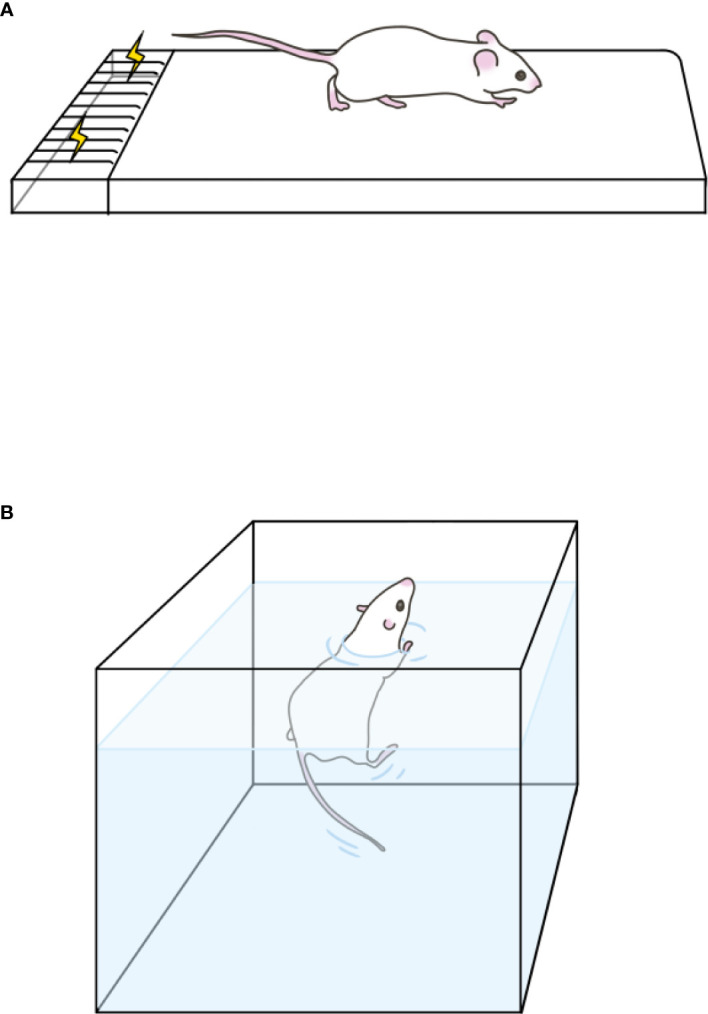
Exercise models. **(A)** Treadmill exercise. It shows one of the runways, the last of which is an electrical grid. **(B)** Swimming training. It is a preheated tank for training.

In experiments involving animals, Sprague-Dawley rats (SD rats) and C57BL/6 mice are typically tested. At different ages, the exercise load borne by the human body is different to some extent; this feature is also reflected in rats and mice (**Figure 1**). Excessive exercise load will affect the physical health of adolescents in the developmental period, most prominently in the bone. Adolescent bone is characterized by more soft tissues and less inorganic salt in bone tissue. Although the adolescent bone has good elasticity, it is harder to bend than fully developed adult bone. Therefore, adolescents cannot bear as much exercise load as adults do. Because the life cycle of experimental animals is shorter than humans, age selection is important in animal research, with researchers generally choosing the most suitable period for exercise.

## Platform model

### Platform model of long-term exercise-induced fatigue in rats

To establish a rat exercise-induced fatigue model, most experiments generally refer to the previous model of exercise program or introduce certain modifications. Among them, researchers mainly select SD rats and Wistar rats to establish models (10, 11). Kim et al. (12) used male SD rats to run at a speed of 20 m/min and 20 min/day in week 1, and 30 min/day at 30 m/min in week 2. The speed started from 12 m/min and increased by 3 m/min every 3 min until it reached 30 m/min, lasting for 60 min. The training scheme designed by Kim et al. (13) paid more attention to the control of exercise load. The 4-week-old SD rats were trained with a gradual load for 6 days a week for 4 weeks, with 20 m/min running for 10 min, 25 m/min running for 20 min, 30 m/min running for 20 min, and 35 m/min running for 30 min, respectively. Finally, the rats ran at 40 m/min to exhaustion. Compared with the above scheme, Xu et al. (14) designed two stages of running training, namely, adaptation and intensive training. The 4-week-old male SD rats were subjected to adaptive running training for five consecutive weeks at the speeds of 15, 22, 27, 31, and 35 m/min for 20 min/day and 5 days a week. For the next 2 weeks, the rats entered high-intensity training and ran at 35 m/min for 20 min/day and 5 days a week. After 7 weeks of training, the rats began from 12 m/min, increased by 3 m/min every 3min, up to 24 m/min, so that the rats ran to exhaustion to determine the exercise tolerance of rats. The characteristics of this exercise program mean that experimental animals can have sufficient time to adapt to the treadmill and high-intensity training.

However, some studies on fatigue take a relatively short period of motion programming. Zhu et al. (15) conducted a 7-day treadmill training for male SD rats, with the speeds of 10, 13, and 20 m/min on the first 3 days, and the speed was increased every 10 min, lasting for 30 min. From the 4th day to the 6th day, the speeds were changed to 18, 22, and 27 m/min; the others were the same as before. The exhaustive exercise test was performed on the 7th day at the speeds of 18, 21, 24, 26, 29, and 34 m/min, increasing every 5 min until exhaustion. Okamura et al. (16) selected 5-week-old male SD rats, and had them run at the speeds of 20, 30, and 35 m/min for 3 days of treadmill training for 20 min each day; on the 4th day, rats ran to exhaustion at a speed of 35 m/min. The exercise-induced fatigue model established by such short-cycle exercise programs pays more attention to the exercise endurance of experimental animals and is accompanied by an exhaustive test. The exercise program with a short exercise cycle may establish a transient exercise-induced fatigue model, but it cannot ensure that the model body is in an irreversible fatigue state. Although the experiment period mentioned above is short, which is conducive to the rapid completion of the experiment, it cannot be applied to all experiments.

In addition to SD rats, many studies have selected Wistar rats to establish models. Liu et al. (17) also designed two stages of adaptive training and intensive training. The first stage was adaptive training lasting for 3 weeks. Male Wistar rats began at 15 m/min and increased every 4 days (to 22, 27, 31, and 35 m/min). Each speed had an adaptive period, including three exercise days at a speed of 20 min/day and 1 day for rest. After resting for 2 days after the adaptive training, rats began 7 days of intensive training, in the first 3 days for 38 m/min, 30 min/day; in the next 2 days for 40 m/min, 35 min/day; and in the last 2 days for 42 m/min, 40 min/day. Xu et al. (14) and Liu et al. (17) both mentioned that exercise programs arranged the speed of adaptive training to increase, but the cycle of adaptive training and the intensity of intensive training were slightly different due to the variety of model rats. Through the comparison of the two programs, we found that the training intensity of Wistar rats was higher than that of SD rats, and the adaptation cycle might be shorter, but the overall exercise cycle was also shorter, so the difference in exercise endurance and the ability of rats of different breeds could not be determined. There are also some scholars that have not designed this part of adaptive training. Dwyer and Browning (18) conducted platform training of 0.8 km/h, 30 min/day, 4 days a week for 6 weeks in 9-week-old male Wistar rats. In the last 2 weeks, the rats ran at a speed of 0.8 km/h for 2 min of exercise test, and then ran at 1.2 km/h, until exhaustion.

The running posture and motor ability of the experimental rats were then observed to judge the degree of fatigue. To sum up, the judgment indexes were based on the following three aspects. First, researchers observed the performance of the rats: depilation, mood, appetite, and response to external stimuli can be used as indicators to judge fatigue. The second was the running posture. When the rat's pedal reflex running posture is not as active as it was at the beginning, strong exercise capacity gradually decreased, indicating fatigue had occurred. Finally, exercise capacity was observed.; If rats could not maintain the original exercise intensity, and the frequency of stimulation was increased or the stimulation time was extended, these external manifestations also helped to determine the fatigue state of rats.

### Platform model of long-term exercise-induced fatigue in mice

Different from rats, the age of mice selected by researchers for exercise-induced fatigue is often older, the selection of exercise training program cycle is different, and the choice of the maximum training speed is also quite different. Ma et al. (19) tested the maximum movement speed of 8-week-old male C57BL/6 mice after three consecutive days of 10 m/min and 15 min/day adaptation training. The mice were pre-adapted at 5 m/min for 3 min and at 10 m/min for 1 min, and then increased by 1 m/min until exhaustion. The mice were then subjected to high-intensity exercise at 85% of their maximum speed for seven consecutive days until exhaustion. Kyung et al. (20) tested 6-week-old male Institute of Cancer Research (ICR) mice for 5 weeks, running at 20, 25, and 30 m/min every week. In week 1 of the three speeds movement, 5 min was allotted for each speed. In week 2, 5 min was allotted for 20 m/min, 10 min for 25 m/min, and 5 min for 30 m/min. In week 3, the exercise times were 5, 15, and 15 min. Finally, in weeks 4–5, the running speed was 30 m/min. The experiment also proved the feasibility of the fatigue model by testing biomarkers. Liu et al. (21) trained 3-month-old male BALB/c mice on a treadmill for 45 min/day, 6 days a week, for 6 weeks. In week 1 for adaptive training, 11 m/min was set as the starting speed for the mice, which was increased by 1 m/min per day. From week 2, the speed increased by 1 m/min per week. After the 6-week experiment, the mice were set to run starting at 15 m/min, increasing to 40 m/min within 3 min for 30 min for three times in a row. The running state of the mice was monitored until the mice failed to adjust their speed with the increase in speed and could not continue running (**Table 1**). 

**Table 1:** Establishment of the treadmill fatigue model.

Species	Age	Sex	Velocity	Duration	Gradient	Reference
SD rats		male	20 m/min in week 1, 30 m/min in week 2; next, speed started from 12 m/min and increased by 3 m/min every 3 min until 30 m/min	20 min/day in week 1, 30 min/day in week 2; the next lasted for 1 h	0°	12
SD rats	4 weeks	male	20 m/min in week 1, 25 m/min in week 2, 30 m/min in week 3, 35 m/min in week 4, 40 m/min to exhaustion	6 days/week, lasting for 4 weeks, 10 min/day in week 1, 20 min/day in weeks 2–3, 30 min/day in week 4	0°	13
SD rats	4 weeks	male	Adaptation training stage: 15, 22, 27, 31, and 35 m/min each week Intensive training stage: 35 m/min Exhaustion: 12 m/min, increased by 3 m/min every 3 min, until 24 m/min	Adaptation training stage: 20 min/day, 5 days/week, lasting for 5 weeks Intensive training stage: 20 min/day and 5 days/week, lasting for 2 weeks	0°	14
Wistar rats		male	Adaptation training stage: began at 15 m/min and increased every 4 days (22, 27, 31, and 35 m/min) Intensive training stage: 38 m/min for days 1–3, 40 m/min for days 4–5, 42 m/min for days 6–7	Adaptation training stage: 20 min/day for 3 days + 1 day for rest, lasting for 3 weeks Intensive training stage: 20 min/day, 5 days/week, 2 weeks; 30 min/day in the first 3 days; 35 min/day in the following 2 days; 40 min/day in the last 2 days	0°	17
Wistar rats	9 weeks	male	0.8 km/h for 6 weeks, the last 2 weeks at 0.8 km/h for 2 min and then run to 1.2 km/h until exhaustion	30 min/day, 4 days/week for 6 weeks	0°	18
ICR mice	8 weeks	male	10 m/min in adaptation training, high-intensity exercise at 85% of their maximum speed	15 min/day in adaptation training, high-intensity exercise until exhaustion	0°	19
ICR mice	6 weeks	male	20, 25, and 30 m/min	week 1 was 5 min; week 2 was 5, 10, and 5 min; week 3 was 5, 15, and 15 min; weeks 4 and 5 within 50 min	0°	20
BALB/c mice	3 months	Male	11 m/min, increased 1 m/min per day in week 1, then increased by 1 m/min per week from week 2	45 min/day, 6 days/week	0°	21

### Swimming fatigue model

At present, swimming is one of the main forms of endurance sports. Endurance exercise usually involves the major muscle groups of the body and lasts for a long time with a certain rhythm. It is characterized by long duration of exercise, large energy consumption, no interval in the exercise, but relatively small exercise intensity. Since the energy required for exercise is mainly derived from the aerobic oxidation system, and the exercise load and oxygen consumption show a linear relationship, therefore, from the perspective of energy metabolism, endurance exercise belongs to the category of aerobic exercise. Swimming as an aerobic exercise is innate to rats and mice. Compared with other exercise methods, rats and mice received less external stimulation during swimming training and were less resistant to training. Therefore, it is more natural to simulate the process of exercise-induced fatigue by swimming.

#### Swimming model of long-term exercise-induced fatigue in rats

Ye et al. (22) prepared a rat model of endurance swimming fatigue, ensuring daily 150 min weight-bearing (about 5% of body weight) swimming for 4 weeks. Zhao et al. (23) conducted 10-day swimming training for male SD rats aged 9–10 weeks. The rats swam for 30 min/day for the first 2 days; in the next 3 days, time increased by 30 min/d to 120 min on day 5, then to 180 min on the days 6 and 7, and in the last 3 days, 180 min training was conducted twice a day, with an interval of 6 h, and the training volume doubled. In this exercise program, the load was not arranged during swimming. As can be seen in the above research, in swimming training for exercise-induced fatigue modeling, single training time is too long. Long single training time is actually more in line with the characteristics of swimming. In the training process, the adjustment of the training intensity of swimming may not be as flexible as that of treadmill exercise. Therefore, when designing the modeling exercise program, researchers often adjust the duration and load of single training, and the long single training time is often relatively large for the regulation of water temperature and the ordeal of the experimenter’s energy. However, the advantage is that swimming can mobilize the whole-body muscles of mice more fully, so there are more muscle groups affected by fatigue.

Tian-Geet al. (24) studied chronic exhaustive exercise using moxibustion resistance. Six- to eight-week-old male SD rats were subjected to adaptive swimming training for three consecutive days, once a day for 20 min, then 3 weeks of weight-bearing (5% of weight) exhaustive swimming exercise. The total exhaustive swimming time of rats (i.e., falling into the water and unable to float for 10 s) was measured. Zhou et al. (25) studied the improvement of long-term fatigue by moxibustion at Shenque (an acupuncture point in Chinese medicine). Male SD rats were placed in a constant temperature water tank for weight-bearing swimming exhaustive exercise (5% of weight), once every other day for 10 days. The posture was observed as an indicator of fatigue in swimming. exercise-induced fatigue was classified as the development stage, when the water reached the rat’s eyes, or the exhaustion stage, when the water reached the tip of the rat’s nose or the rat sank. Therefore, to establish a standard fatigue model, the exercise state of rats should be observed.

#### Swimming model of long-term exercise-induced fatigue in mice

To achieve the effect of overtraining, increasing the load of the exercise program is often used to establish the exercise-induced fatigue model of swimming training. Zhang et al. (26) made male ICR mice aged 6–8 weeks undergo swimming adaptation training for 1 week, swimming for 30 min and 45 min, respectively in the first 2 days, and swimming for 60 min in the last 4 days. From weeks 2 to 6, mice were trained with weight increasing by 2% per week until 10% of their body weight was reached. Chi et al. (27) performed weight-bearing (5% body weight) or weight-free swimming training on 2-month-old male Kunming mice, 5 days a week for 4 weeks. Similar to Zhang et al., Lin et al. (28) conducted adaptive training for male ICR mice aged 4–6 weeks for 1 week, swimming for 30 min and 45 min on the first 2 days and 60 min on the last 3 days. Then, from weeks 2 to 6, the mice were subjected to swimming training with weight-bearing of 10% of their body weight for 60 min, emphasizing weekly monitoring of their weight and timely adjustment of weight-bearing. Yeh et al. (29) conducted 6-week swimming training on 4-week-old male ICR mice. Week 1 of the adaptive training program was consistent with Lin et al. (28). From weeks 2 to 6, swimming time was also 60 min/day, but the initial load was less and gradually increased during training. Weight-bearing was 1% of the body weight in the second week, 2% in weeks 3–4, and 3% in weeks 5–6. Finally, 5% of the body weight was used to swim to exhaustion to evaluate the endurance of mice.

Long-term exercise-induced fatigue animal models mainly use treadmill and swimming exercise. In treadmill exercise, the intensity is changed by adjusting the slope, load, speed, and time until fatigue is reached. Resistance exercise such as weight-bearing climbing exercise has also been used to model exercise-induced fatigue; however, it is speculated that this exercise model is less common due to the determination of exercise-induced fatigue being relatively vague and only for the fatigue caused by resistance movement. In addition, the equipment required for the experiment is not common, making it difficult to implement.

In **Table 2**, we summarized the different ways to induce exercise-induced fatigue in animal models. There are many advantages to treadmill training for v. The main advantages of experimental animals using treadmill exercise are as follows: the exercise mode is in line with the daily exercise of experimental animals; compared with other sports, the exercise load and training intensity can be regulated more accurately by manual control of the speed and slope, in treadmill exercise; and animals in each channel have independent movement, are not disturbed, and have less restrictive factors between each other. However, animal treadmill generally adopts electric shock, sound stimulation, and brush stimulation to drive animals to run. When the speed of the animal is lower than the speed of the treadmill, it will be stimulated to force it to complete the exercise at the specified strength and time, so animals are prone to defensive reflex stress and mechanical damage. The advantages of modeling by swimming are that the experimental equipment is simple and inexpensive and animals are less stimulated outside of the exercise in water, and the method is relatively mild. However, there are also certain shortcomings: Each animal will have individual differences in fatigue, and some can swim for hours; during the experiment, it is necessary to control the water temperature at all times, maintain constant temperature, and consume manpower; the workload of the experiment is large; and the experimental animals should be dried after swimming, such as by blow-drying and wiping.

**Table 2:** Establishment of the swimming fatigue model.

Species	Age	Sex	Duration	Weight-bearing	Reference
SD rats	8 weeks	male	Once a day for 3 h days 1–7 and twice a day for 6 h days 8–10	None	22
SD rats	9–10 weeks	male	30 min a day, days 1–2; days 3–5, increase 30 min a day until day 5 is up to 120 min	None	23
SD rats	6–8 weeks	male	20 min per day for 3 days, 3 weeks to exhaustion	5% of body weight	24
SD rats		male	Once every other day for 10 times	5% of body weight	25
ICR mice	6–8 weeks	male	Adaptation training for 1 week, 6 days/week, 30 and 45 min in days 1 and 2, 1 h in the following 4 days and the following training weeks	No weight for week 1; weeks 2–6, 2% of body weight per week until 10% is reached	26
Kunming mice	2 months	male	6 days a week, 60 min a day for 4 weeks	One group for 5% of body weight; one group, no load	27
ICR mice	4–6 weeks	male	Adaptation training for 1 week, 5 days/week, 30 and 45 min in days 1 and 2, 1 h in the following 3 days and the following training weeks	No weight for week 1; weeks 2–6, 10% of body weight	28
ICR mice	4 weeks	male	Adaptation training for 1 week, 30 and 45 min in days 1 and 2, 1 h in the following 3 days and the following training weeks	No weight for week 1, 1% in week 2, 2% in weeks 3–4, 3% in weeks 5–6. Finally, 5% of body weight was used to swim until exhaustion	29

## Effects of long-term exercise-induced fatigue on multiple organ phenotypes

The successful establishment of the exercise-induced fatigue model is only the beginning of research. On this basis, it is particularly important to observe the multiple organ phenotypes. It allows the identification of whether the fatigue model is successful or not. It can also give some guidance to exercise monitoring and fatigue recovery by synthesizing the possible pathological phenomena after exercise-induced fatigue. We reviewed phenotypes of long-term exercise fatigue (e.g., nervous system damage, myocardial cell damage, bone mineral density changes, and skeletal muscle damage).

### Nervous system phenotype

#### Central nervous system

The cerebral cortex controls various functional activities of the body, such as movement, sensation, and language, and performs the corresponding regulation of the central nervous system. Long-term exercise-induced fatigue disrupts physiological systems and the dynamic balance of the body. Fatigue caused by exercise will affect cognitive function (30). Mental fatigue can be defined as a psychobiological state caused by long-term fatigue that has the potential to reduce cognitive performance and exercise performance. Recent studies have clearly shown that brain catecholamines are related to the occurrence of fatigue during endurance exercise (31). Evidence provided that the norepinephrine neurotransmitter system accelerated central fatigue, consistent with a faster increase in perceived fatigue scores (31). Brain nerve transmission is also considered to play an important role in mental fatigue. The central catecholamines, dopamine, and norepinephrine, are thought to play a principal role in central fatigue and have been implicated in the onset of mental fatigue and its effects on subsequent physical or cognitive performance. Several neurotransmitter systems may be related to multiple brain regions, such as the anterior cingulate cortex. Prolonged mental exertion could induce adenosine accumulation in the anterior cingulate cortex, leading to a higher-than-normal perceived effort during a subsequent endurance exercise. The sum of these changes may explain the damaged state of endurance performance during mental fatigue (32).

After long-term exercise-induced fatigue, mice showed behavioral abnormalities such as bradykinesia, rigidity, incoordination, and posture imbalance. The cortical striatal nerve pathway, as the key signal pathway of the extrapyramidal system of the brain regulates synaptic transmission in the nervous system, may affect cognitive function. Wang et al. (33) observed the ultrastructural changes of asymmetric synapses in rat striatum after repeated exercise-induced fatigue by transmission electron microscope. After repeated exercise-induced fatigue, the levels of synaptic active region proteins Munc13 and RIM1, synaptic vesicle protein Rab3A, and postsynaptic density PSD-95 protein in striatum of rats were found to be abnormal. The abnormal changes in synaptic ultrastructure and related protein levels of these asymmetric cortical striatum may be the structural basis for the plasticity damage of cortical striatum after exercise-induced fatigue.

The potential mechanism of fatigue seems to depend on the neuroinflammation pathway. Fatigue not only occurs in chronic and acute diseases, but also occurs in long-term intense exercise. The level of cortisol and other hormones is upregulated during long-term intense exercise, which changes the activity of neurons and induces an increase of the anti-inflammatory cytokines IL-10, IL-1 receptor antagonist, and soluble TNF receptors. The abnormal regulation of the peripheral-central nervous system interface is also related to fatigue (34).

#### Peripheral nervous system

Spinal motor neurons regulate the discharge level of motor units and the contraction state of muscles through the integration of advanced central down-transmission information and peripheral afferent information. Spinal cord 5-hydroxytryptamine (5-HT) is involved in the occurrence of exercise-induced fatigue and has a direct regulatory effect on motor neurons, which can cause its excitation (35). After exercise-induced fatigue, there is a decrease in 5-HT in the anterior horn of the spinal cord, the excitability of motor neurons, the recruitment of motor neurons, and the motor output facilitation. These changes are related to fatigue acceleration (35). In addition to its direct effects on behavior, 5-HT can modulate fatigue through changes in regulation of body temperature. 5-HT and dopamine are neurotransmitters related to fatigue, which may lead to reduced or interrupted exercise intensity. The increase of 5-HT activity in rodents during exercise may reduce exercise performance, while the increase of dopamine activity is related to the improvement of exercise performance (35).

Tanaka et al. (36) used load forced swimming to establish an exercise-induced fatigue model and found that rats kept in the wet cage for 5 days showed a reduction in 2-[18F]fluoro-2-deoxy-D-glucose uptake into their brain; after 1 day of session, the ratios of 5-hydroxyindoleacetic acid (5-HIAA)/5-HT and [3,4-dihydroxyphenyl-acetic acid (DOPAC)+homovanillic acid (HVA)]/DA in all brain regions increased significantly; the 5-HIAA/5-HT ratio in the hippocampus and hypothalamus and (DOPAC+HVA)/DA ratio in the striatum and hypothalamus recovered after 5 days of the sessions. It is suggested that decreased glucose intake and insufficient serotonin and dopamine turnover caused by deprivation of rest are associated with central fatigue. However, human experiments, especially those involving nutritional supplementation or pharmacological operations, have produced contradictory results in the relationship between serotonin, dopamine, and fatigue. The only clear and repeatable effect observed in humans is that performance in high temperature environments is improved after treatment with dopamine reuptake inhibitors.

However, 5-HT and dopaminergic systems are thought to inhibit each other, and the ratio of 5-HT to dopamine seems to determine fatigue better than merely analyzing or manipulating one of these two transmitters. 5-HT stimulation and the DA inhibition of the release of prolactin by anterior pituitary lactate cells are used to determine the activity of neurotransmitters in the central nervous system, and the increase in concentration of prolactin indicates that 5-HT activity increases and/or dopamine activity decreases, and that long-term exercise-induced fatigue will lead to higher 5-HT concentration. In addition, 5-HT receptor sensitivity can regulate fatigue during long-term exercise (35). Zhu et al. (37) found that verbascoside was as effective as caffeine, and 10 mg/kg of verbascoside could improve the exercise endurance of rats, inhibit the increase of exercise-induced 5-HT synthesis and the expression of the tryptophan hydroxylase (TPH2) protein, and prevent the decrease of the exercise-induced serotonergic type 1B inhibitory autoreceptors (5-HT1B) protein expression in caudate putamen. It suggested that the anti-fatigue mechanism of verbascoside might be related to the inhibition of the synthesis of 5-HT and TPH2 expression in the caudate putamen of rats induced by exercise and the increase of 5-HT1B expression.

### Cardiac phenotype

Within the cardiovascular system, exercise intensity and duration can cause human cardiac function and biochemical disturbance. Severe endurance exercise can cause transient functional and biochemical cardiac disturbances lasting 24–48 h. The amplitude and duration of ventricular function decline and cardiac injury marker increase caused by exercise are affected by exercise intensity and duration (38). Long-term strenuous exercise may lead to adverse waves and structural remodeling in the normal heart (39). In high-intensity endurance exercise, some athletes may have right ventricle (RV) cardiomyopathy due to genetic susceptibility, while others may have arrhythmia in the RV. Long-term intensive exercise training can lead to fibrosis in animal models. In athletes, myocardial fibrosis is closely related to long-term endurance exercise (40). Endurance exercise increases the volume and pressure load of the two ventricles and increases myocardial mass. The degree of volume increase and changes in myocardial structure lead to impaired right ventricular function (40). High-intensity endurance exercise does not affect the left ventricle (LV) volume or function, but it will lead to RV expansion and a decrease in RV ejection fraction, which becomes more significant during exercise. At the same time, right ventricular–arterial coupling changes suggest that this may be exercise-induced right ventricular systolic dysfunction (41). Severe endurance activities can cause a particularly high pressure on RV, which may lead to an arrhythmia-promoting status similar to right or (less commonly) left ventricular cardiomyopathy. It is suggested that “exercise induced arrhythmia RV cardiomyopathy” may be the result of excessive RV wall pressure during exercise (42). In addition, a meta-analysis also concluded that long-term endurance exercise was associated with a significant reduction in RV function, while left ventricular function was relatively unaffected (43). It is suggested that future studies should examine the potential clinical consequences on RV during exercise.

Long-term overtraining can affect normal function, such as heart response. During intense exercise, the heart rate is gradually accelerated, the diastolic period of the heart is gradually shortened, and the blood perfusion time of the myocardium is shortened. These responses lead to myocardial ischemia, hypoxia, and myocardial micro-structure damage (44). Long-term exercise-induced fatigue causes myocardial cell apoptosis, which leads to abnormal morphological structure of myocardial cell nucleus and increased apoptosis rate. Excessive apoptosis of myocardial cells affects the overall heart structure and function, and even causes sudden cardiac death (45). Tuo et al. (46) evaluated the changes of myocardial morphology, injury indexes, and inflammatory-related proteins in overtraining rats, and found that astragalus polysaccharides significantly increased the cell viability of H9c2 cells, reduced the apoptosis of myocardial cells, and reduced myocardial injury related indicators. The apoptotic index and number of cardiomyocytes increased significantly. There was also an increase in B cell lymphoma/leukemia-2 and Bcl-2 associated X proteins within the myocardial tissues. Myocardial cells were damaged, and the number of myocardial cell apoptosis was significantly reduced. Overall, the heart structure and function were affected, ultimately leading to sudden cardiac motor death.

### Bone phenotype

Long-term high-intensity training is detrimental to bone health. The periodicity of overload exercise will lead to damage of bone microstructure. Most studies have shown that exercise-induced fatigue can cause bone mineral density (BMD) decrease and bone loss. Kathrin et al. (47) found that the use of the treadmill’s maximum speed of 80% with a training-intensity slope of 10° led to the trabecular bone significantly decreasing. Li et al. (48) found that after 8-week high-intensity running at a rate of 26.8 m/min, a slope of 10°, each time for 60 min, the BMD of the subchondral plate and trabecular bone of rats increased abnormally, and the porosity decreased, resulting in brittle and rigid subchondral bone affecting the articular cartilage. Hind et al. (49) found that weekly running distance was negatively correlated with lumbar BMD, and long-distance running led to a large amount of energy consumption, resulting in insufficient energy and reduced bone formation, which was positively correlated with low BMD. If energy intake is insufficient, long-term long-distance running increases energy deficiency, resulting in low BMD. It can be seen from animal experiments that exercise-induced fatigue has a significant negative impact on bone mass, but there seems to be a lack of relevant research on bone strength. Polisel et al. (50) found that BMD, maximum femur load, elasticity, stiffness, and fracture displacement of high-intensity interval training in mice decreased five times a week for 10 weeks, but there was no significant difference, while the toughness of bone decreased significantly, suggesting a risk of fracture. exercise-induced fatigue also affects bone metabolism. Chen et al. (51) found that rats performing long-term high-load and high-repeat tasks (upper limb stretching and lever pulling task) led to fatigue, cortical bone thinning in the distal radius, and increased bone cell apoptosis, resulting in decreased sclerostin (SOST) upregulated osteoblasts. In addition, bone cell apoptosis promotes the release of nuclear factor-κB receptor activator ligand (RANKL), increases osteoclasts, and promotes changes in bone catabolism. As such, it is important to consider the adverse effects of excessive physical exercise on bone health.

### Skeletal muscle phenotype

The occurrence of exercise-induced skeletal muscle injury is closely related to the change of mitochondrial function in skeletal muscle cells. Research focuses on mitochondrial dysfunction induced by heavy load and/or long-term exercise. Liu et al. (21) found that long-term exercise-induced fatigue significantly increased oxidative stress indexes in the skeletal muscle and serum and reduced the total antioxidant content in skeletal muscle and blood. These changes may lead to the accumulation of free radicals and induce muscle injury and fatigue. Mitochondrial respiratory chain complex activity is closely related to mitochondrial function.

Skeletal muscle mitochondrial respiratory chain activity is also affected by long-term exercise-induced fatigue. Ping et al. (52) found that exercise-induced fatigue could induce muscle structural damage, enzyme abnormality, respiratory function decline, and mitochondrial downregulation. Abnormal mitochondrial structure after weight-bearing exercises leads to dysfunction affecting skeletal muscle function, which leads to muscle injury through subsequent reactions.

We summarized the related phenotypes of some human systems and found that exercise-induced fatigue causes irreversible damage to muscles and organs (**Table 3**). The fatigue monitoring of athletes is often carried out through the decline in their sports performance and some simple physiological manifestations, and further detection is carried out after finding problems. The next section provides a more comprehensive idea for daily monitoring through the phenotypic changes of each system after exercise-induced fatigue, so as to be able to quickly find the fatigue state of athletes and then give recovery measures.

**Table 3:** Effects on multiple organ phenotypes.

System	Organ (Classification)	Phenotype	Reference
Nervous system	Central nervous system	exercise-induced fatigue leads to the plasticity damage of cortical striatum and a significant decline in learning and memory behavior; affects cognitive function; induces the gene expression of pro-inflammatory cytokines	30–34
Nervous system	Peripheral nervous system	5-HT in the anterior horn of the spinal cord decreased; the excitability of motor neurons decreased; the recruitment of motor neurons decreased; the motor output lost facilitation	35–37
Cardiovascular system	Heart	Myocardial ischemia and hypoxia, myocardial microstructure damage; adverse waves and structural remodeling; myocardial fibrosis; calcium balance disorder in myocardial cells; myocardial cell apoptosis, abnormal morphological structure of myocardial cell nucleus	38–46
Locomotor system	Bone	Reduces BMD and causes bone loss; damages the bone microstructure; increases bone cell apoptosis; decreases osteoblasts and increases osteoclasts; is not conducive to bone health	47–51
Locomotor system	Skeletal muscle	Muscle injury; mitochondrial dysfunction of the skeletal muscle; the activity of skeletal muscle mitochondrial respiratory chain is affected	52, 53

## Indicators of long-term exercise-induced fatigue monitoring

It is important to monitor exercise-induced fatigue as it causes damage to muscles and organs and negatively impacts motor performance. In this section, we summarized the key indicators of exercise-induced fatigue.

### Exhaustion time

The exercise endurance of the experimental animals was measured from the beginning of the exercise up to the time or onset of exhaustion. The exhaustion of the swimming model was based on drowning time (i.e., 10 s and did not appear on the water surface for three consecutive seconds). The exhaustion of the treadmill model can be manifested as the failure of animals to adhere to the original running speed at the end of exercise, and more than three times at 1/3 of the runway after being stuck successively, and the stimulation drive is ineffective. The signs after running were shortness of breath, burnout, the gap between the abdomen and runway, and slow response to stimulation; usually, these signs were used to determine the onset of fatigue and then the time is recorded. While exercise-induced fatigue will reduce exhaustion time, the following studies show that anti-fatigue drugs can effectively improve exhaustion time. Lima et al. (53) defined the effect of ibuprofen on exercise-induced fatigue by using exhaustion time. Yin et al. (54) studied the anti-fatigue and vascular protective effect of quercetin-3-O-gentiobiose (QG) on oxidative stress and vascular endothelial dysfunction induced by endurance swimming in rats. Through the establishment of load swimming fatigue model, the exhaustive swimming time was measured to determine the exercise tolerance of rats. Physical fatigue was related to long-term swimming exercise. It was found that ginsenoside Rb1 and QG prolonged the swimming time, and QG had an anti-fatigue effect. In conclusion, exhaustion time as an indicator is mostly used to test the anti-fatigue effect of drugs. Long or increased exhaustion time corresponds to better exercise capacity and endurance.

### Organ index

Organ index refers to the percentage of organ weight in body weight. For example, in mice, organ index = organ weight of mice/body weight of mice × 100%. This is an important indicator reflecting the internal organs and nutritional status of experimental animals. Studies have found that exercise-induced fatigue is often accompanied by muscle and organ damage (21, 52). If organ edema or congestion occurs, organ index will increase; if the organ index decreases, it can indicate organ atrophy and other degenerative changes (55). However, the limitation of this indicator is that animal weight changes are sometimes much larger than most organ weight changes. Different animal strains, age, and other external factors usually affect the determination of this index, so this index is less used in experiments. In addition, Hsu et al. (56) believed that fatigue may lead to a decline in the index of human organs, affecting the daily life and sports performance of ordinary people and athletes.

### Biochemical indicators

#### Lactic acid

Intensive exercise consumes a large amount of ATP and creatine phosphate (CP). When the two are insufficient in the body, the body will use anaerobic glycolysis to produce lactic acid (LA) for energy. LA usually increases linearly with exercise intensity. When the intensity exceeds a certain range, the serum LA content increases exponentially. Increased LA content is not related to age, gender, and physical condition. However, LA detection has limitations, which depend on ambient temperature, drinking water, diet, LA clearance rate, and glycogen content (57). These factors may interfere with the results of the experiment.

In the 1980s, Karlsson found that the generation of exercise-induced fatigue was related to the increase of LA after exercise. In particular, fatigue is related to muscle LA dissociation of hydrogen, leading to a decreased pH value. The decreased pH value affects the calcium-binding ability of myosin, and the activities of creatine kinase, ATPase, phosphofructose kinase, and other kinases, thus affecting the metabolism of the LA system. LA and its reduced pH value are two of the commonly used biomarkers in exercise-induced fatigue or exhaustive exercise.

#### Urea nitrogen

Blood urea nitrogen (BUN) is the main product of human protein and amino acid metabolism, which is positively correlated with physical function, degree of fatigue, and load. Fatigue makes glycogen and fat metabolism inadequate to meet energy needs, so the body must rely on protein decomposition to provide energy. BUN reflects the metabolic intensity of amino acids in the body and is usually used as an indicator to evaluate exercise. Intensive exercise strengthens the decomposition and metabolism of proteins and amino acids, and increases the BUN content in the body (58). Duan et al. (59) studied the anti-fatigue effect of luteolin-6-C-neohesperidin (LN) on oxidative stress injury using a swimming model in rats. LN intervention prevented the release of BUN in a dose-dependent manner. LN activated oxidative stress and inflammatory factors and significantly improved the endurance of forced swimming in rats, thus indicating improvements on fatigue-related changes. Lee et al. (60) found that the high-dose group of perch essence could reduce the serum BUN level of mice after swimming endurance training. In summary, BUN can be used as a biomarker for evaluating exercise-induced fatigue.

#### Malondialdehyde

Malondialdehyde (MDA) is the degradation product of polyunsaturated fatty acid peroxides, which is the product of free radicals acting on lipid peroxidation. MDA content can reflect the severity of the free radical attack and damage on cells. The plasma analysis of marathon athletes by Child et al. (61) confirmed that MDA content increased significantly after exercise. In rats, Kazim et al. (62) evaluated fatigue time and body weight and composition to study the effects of Carnipure tartaric acid supplementation on endurance, recovery, and fatigue. Fifty-six Wistar rats were divided into groups. The exercise group received an exercise program and MDA level was measured. In the exercise rats with Carnipure Tartrate supplementation, serum MDA concentration decreased. Carnipure Tartrate supplementation during exercise was also beneficial to exercise performance, recovery, and fatigue, and improved lipid distribution and antioxidant capacity. exercise-induced fatigue can increase MDA, which may react physiologically with several nucleosides to form the adducts of deoxyguanosine and deoxyadenosine. The increased exercise intensity may increase purine oxidation, resulting in an increase in the formation of uric acid. Some anti-fatigue drugs affect the content of MDA through the adaptive mechanism of oxidative stress.

#### Superoxide dismutase

Superoxide dismutase (SOD) is an important anti-peroxidase in the free radical scavenging system. The activity of SOD represents the content of free radicals in the body. When exercise-induced fatigue causes the content of free radicals to be high, SOD enzyme activity is also high. After long-term exercise, plasma MDA in plasma and SOD are significantly increased. SOD can reflect the generation and elimination rate of free radicals in vivo, changes in free radical metabolism, and the degree of exercise-induced fatigue of the body (63).

Shui et al. (64) developed metabolomic methods to study the anti-fatigue mechanism of Yiguanjian (YGJ), a type of traditional Chinese medicine. They used gas chromatography–mass spectrometry and multivariate statistical techniques to estimate the degree to which YGJ alleviated swimming fatigue in mice. Biochemical indexes such as SOD, BUN, and LA were measured. In fatigued mice, SOD decreased while BUN and LA increased. These changes were reversed after YGJ treatment. SOD is one of the defense lines of the antioxidant enzyme system, which can eliminate free radicals generated in physical exercise and reduce body pressure and fatigue. In this experiment, the significantly increased SOD activity in the high-dose group suggested that YGJ is an antioxidant that can reduce oxidative stress caused by exercise-induced fatigue.

#### Testosterone

Testosterone is secreted by the male testis or the female ovary, and the adrenal gland also secretes a small amount of testosterone. Testosterone maintains muscle and bone strength and quality and enhances physical fitness. Studies have shown that long-term high-intensity exercise or excessive training can decrease blood testosterone. Cadegiani and Kater (65) found that the serum testosterone level of athletes with overtraining syndrome was significantly lower than that of healthy athletes, even similar to that of healthy sedentary groups. Urhausen et al. (66) found that the testosterone levels of overtraining endurance athletes decreased significantly. The monitoring results of the above human experiments suggested that after the occurrence of exercise-induced fatigue, physical fitness decreased, and exercise ability was negatively affected, resulting in the decrease of serum testosterone level. Therefore, testosterone can be used as a monitoring indicator of exercise-induced fatigue.

#### Glutathione peroxidase

Glutathione peroxidase (GSH-Px) catalyzes the reduction of H2O2 and protects the integrity of cell membrane structure. GSH is another substrate of the GSH-Px reduction reaction. Studies suggest that exercise-induced fatigue increases GSH-Px activity. Excessive exercise can cause GSH-Px activity in muscle tissue to increase. Li et al. (67) found that essential oils can alleviate exercise-induced fatigue in rats caused by swimming, and increase their activity of GSH-Px. Kazim et al. (62) studied the effects of Carnipure Tartrate on fatigue recovery by evaluating fatigue time and body composition in rats. The activities of antioxidant enzymes such as GSH-Px were measured. The results showed that the levels of antioxidant enzymes in rats with exercise and Carnipure Tartrate supplementation were significantly increased in a dose-dependent manner, indicating that the antioxidant capacity of rats was improved. It was concluded that Carnipure Tartrate supplementation during exercise was beneficial to exercise performance and fatigue recovery and improved antioxidant capacity.

#### Cortisol

Cortisol is a glucocorticoid hormone synthesized and secreted by the bundle of adrenal cortex. Its main function is to increase gluconeogenesis. It can also promote protein and fat metabolism significantly. After various sports, cortisol levels increase.

exercise-induced fatigue causes various disturbances of dynamic balance and damage of biological energy related to frequent and intense muscle activities. Stajer et al. (68) found that there was a moderate to strong positive linear correlation between the change of serum cortisol induced by exercise-induced fatigue and the level of serum guanidine acetic acid, indicating that cortisol was related to the damage of biological energy caused by intense exercise. The indicative effect of cortisol on exercise-induced fatigue was used to test the on-site performance of athletes. Schmikli et al. (69) tested athletes on-site whose performance declined during the season and found that the cortisol level was low. Mechanical massage was used to intervene in exercise-induced back muscle fatigue, and the serum cortisol level was significantly decreased after intervention (70). Cortisol can be used as a biomarker of exercise-induced fatigue.

#### Creatine kinase

Serum creatine kinase (CK) is a high-energy phosphate transferase for energy supply in skeletal muscle contraction. It can catalyze the reversible transfer of high-energy phosphate bonds. During exercise, it is released intracellularly. Therefore, its content can reflect the degree of skeletal muscle injury. It is positively correlated with the degree of muscle injury, which directly affects the aerobic and anaerobic metabolism ability of the body during exercise (71). Therefore, it is considered an important indicator to measure exercise-induced fatigue.

After over-distance marathon running and weight-bearing downhill running, skeletal muscle cells were damaged, resulting in a significant increase in total serum CK activity and an increase in CK activity (72). The Ironman Triathlon is a high-intensity and long-term form of exercise. Huang et al. (73) found that the CK index of athletes who intake Lactobacillus plantarum PS128 significantly decreased during the recovery stage, suggesting that it may contribute to inflammation or oxidative regulation. In the experiment, CK index was used as one of the biochemical indicators to measure fatigue recovery. Mechanical massage was used to intervene in exercise-induced back muscle fatigue. There was no significant difference in creatine kinase level immediately after intervention, but there was significant difference in creatine kinase level 24 h later between the two groups of control and treatment (P<0.05) (70).

We sorted out the relevant indicators that can reflect the exercise-induced fatigue model to some extent, mainly some biochemical indicators. However, there is a lack of a standard for the selection of indicators. At present, most animal experiments only select several of them, and most of them are in vitro experiments, lacking the experience and technology of in vivo monitoring (**Table 4**).

**Table 4:** Monitoring indicators of exercise-induced fatigue.

**Indicator**	**Variation**	**Significance**	**Reference**
Exhaustion time	↓	Records the time from the start of the exercise to the onset of exhaustion, reflecting the intuitive performance of exercise capacity	54,55
Organ index	↓	The percentage of organ weight in body weight; an important indicator reflecting the internal organs and their nutritional status	56,57
Lactic acid (pH value)	↑(↓)	One of the products of anaerobic respiration; its pH value affects the metabolism of LA system	58
Blood urea nitrogen	↑	The main end product of human protein and amino acid metabolism, a reflection of the metabolic intensity of amino acids in the body	59–61
Malondialdehyde	↑	The degradation product of polyunsaturated fatty acid peroxides; the reflection of the severity of free radical attack and damage on cells	63
Superoxide dismutase	↑	An important anti-peroxidase in free radical scavenging system; an antioxidant that can reduce oxidative stress caused by exercise-induced fatigue	64,65
Testosterone	↓	Maintains muscle strength and quality; maintains bone density and strength	66,67
Glutathione peroxidase	↑	Catalyzes the reduction of H_2_O_2_; protects the integrity of cell membrane structure	64,68
Cortisol	↑	Increases gluconeogenesis, protein, and fat metabolism; is related to the damage of biological energy caused by intense exercise	69–71
Creatine kinase	↑	The reflection of the degree of skeletal muscle injury; it is positively correlated with the degree of muscle injury, which directly affects the body’s aerobic and anaerobic metabolism during exercise	71–73

↑: up; ↓: down.

## Summary

Exercise-induced fatigue is a research hot spot. As a branch of exercise-induced fatigue, long-term exercise-induced fatigue on the body is different from short-term exhaustion, which cannot be ignored. The most obvious influence of long-term exercise-induced fatigue in competitive sports is the decline in sports performance. This review summarizes how the two most commonly used training methods, treadmill exercise and swimming exercise, were used to summarize the different exercise schemes of the fatigue models of different strains of large mice. There is still a lack of a unified standard for designing exercise schemes in sports. However, after summarizing the advantages and disadvantages of these two sports methods, the modeling scheme summarized in this paper can be used or improved according to the researchers’ needs.

To establish a good animal model of exercise-induced fatigue, we must pay attention to and control the variables and exclude interference factors. As a control, researchers should pay attention to external temperatures, humidity, details of the intervention, or the strain and age of the animal, and select the appropriate exercise scheme on the premise that these factors are noted. However, there are still some problems in the establishment of fatigue models for experimental animals to simulate the exercise-induced fatigue experienced by athletes. For example, the movement habits of rats tend to prefer a dark cycle, meaning more activities in the evening, raising the question of whether the choice of exercise time in the future can be considered and controlled. The premise of establishing an ideal exercise-induced fatigue model is to discern exercise-induced fatigue. Through the observation of multiple organ phenotypes and monitoring various indicators, we can determine whether a model is successfully established. Similarly, it can provide reference when implementing exercise plans for athletes.

This paper mainly summarizes the research on animal fatigue models rather than humans, mainly because animals are easier to control than humans if researchers want to further explore a certain anti-fatigue substance or biochemical marker. In fact, the information obtained from animal research and athlete training is interchangeable. Changes in indicators or organ phenotypes found from animals can be used to monitor humans to predict the occurrence of fatigue and avoid it. Similarly, signs of suspected fatigue found from athletes can be verified by animal studies and solutions can be proposed.

Athletes’ performance is closely related to the body’s functional state. If there is long-term exercise-induced fatigue, it will affect the athletes’ performance and cause organic damage. Monitoring athletes after transient exercise-induced fatigue to ensure recovery is conducive to improving exercise capacity and breaking through their own threshold. For coaches, it can provide a theoretical basis for optimizing training programs and improving overall team performance.

## Author contributions

LZ wrote the brief introduction of this article. KY, HG, XL, ZZ, and LZ were responsible for writing the manuscript. BG revised the manuscript. All authors approved the final version of this manuscript.

## Acknowledgments

We appreciate the time and effort of the participants in this study. The work was supported by the National Natural Science Foundation of China (82172475); Shanghai Frontiers Science Research Base of Exercise and Metabolic Health, Shanghai University of Sport, Shanghai 200438, China; Shanghai Key Lab of Human Performance (Shanghai University of sport) (11DZ2261100).

## Conflict of interest

The authors declare that the research was conducted in the absence of any commercial or financial relationships that could be construed as a potential conflict of interest.

## Publisher’s note

All claims expressed in this article are solely those of the authors and do not necessarily represent those of their affiliated organizations, or those of the publisher, the editors and the reviewers. Any product that may be evaluated in this article, or claim that may be made by its manufacturer, is not guaranteed or endorsed by the publisher.
